# Amino-Functionalized Titanium Based Metal-Organic Framework for Photocatalytic Hydrogen Production

**DOI:** 10.3390/molecules27134241

**Published:** 2022-06-30

**Authors:** Niannian Hu, Youlie Cai, Lan Li, Xusheng Wang, Junkuo Gao

**Affiliations:** 1 Institute of Functional Porous Materials, School of Materials Science and Engineering, Zhejiang Sci-Tech University, Hangzhou 310018, China; 202030302117@mails.zstu.edu.cn (N.H.); 201920301012@mails.zstu.edu.cn (Y.C.); 2 College of Materials Science and Engineering, China Jiliang University, Hangzhou 310018, China; lilan123@mail.ustc.edu.cn; 3 Zhejiang Provincial Innovation Center of Advanced Textile Technology, Shaoxing 312000, China; 4 College of Chemistry and Materials Science, Jinan University, Guangzhou 510632, China

**Keywords:** metal-organic frameworks, photocatalytic hydrogen production, amino-functionalized, titanium, photocatalyst

## Abstract

Photocatalytic hydrogen production using stable metal-organic frameworks (MOFs), especially the titanium-based MOFs (Ti-MOFs) as photocatalysts is one of the most promising solutions to solve the energy crisis. However, due to the high reactivity and harsh synthetic conditions, only a limited number of Ti-MOFs have been reported so far. Herein, we synthesized a new amino-functionalized Ti-MOFs, named NH_2_-ZSTU-2 (ZSTU stands for Zhejiang Sci-Tech University), for photocatalytic hydrogen production under visible light irradiation. The NH_2_-ZSTU-2 was synthesized by a facile solvothermal method, composed of 2,4,6-tri(4-carboxyphenylphenyl)-aniline (NH_2_-BTB) triangular linker and infinite Ti-oxo chains. The structure and photoelectrochemical properties of NH_2_-ZSTU-2 were fully studied by powder X-ray diffraction, scanning electron microscope, nitro sorption isotherms, solid-state diffuse reflectance absorption spectra, and Mott–Schottky measurements, etc., which conclude that NH_2_-ZSTU-2 was favorable for photocatalytic hydrogen production. Benefitting from those structural features, NH_2_-ZSTU-2 showed steady hydrogen production rate under visible light irradiation with average photocatalytic H_2_ yields of 431.45 μmol·g^−1^·h^−1^ with triethanolamine and Pt as sacrificial agent and cocatalyst, respectively, which is almost 2.5 times higher than that of its counterpart ZSTU-2. The stability and proposed photocatalysis mechanism were also discussed. This work paves the way to design Ti-MOFs for photocatalysis.

## 1. Introduction

Photocatalytic hydrogen production from water using solar light as clean and sustainable energy is one of the most promising solutions to solve the energy crisis [1,2,3,4,5]. As a new kind of porous materials, metal-organic frameworks (MOFs) have been applied in many fields such as gas adsorption/storage/separation, sensor, drug delivery, batteries, electrocatalysis, photocatalysis, due to their ultrahigh surface area and void space, adjustable structure, tunable pore sizes, and modifiable internal surfaces [6,7,8,9,10,11,12,13,14,15,16,17,18,19,20,21]. Since Mori et al. first reported that MOFs can achieve photocatalytic hydrogen production, a series of MOFs have been reported to show potential in this application [22,23,24,25,26,27,28,29]. However, most of those MOFs survived low stability during photocatalysis process.

Titanium-based MOFs (Ti-MOFs), as a kind of robust MOFs, are constructed by organic ligands and high valent Ti^4+^ ions, showing high chemical stability [9,30]. The high stability of Ti-MOFs can be explained by the Pearson’s hard-soft acid-base principle, in which carboxylate ligands can be seen as hard base, and the high valent Ti^4+^ ions as hard acid, thus, robust coordination bond between carboxylate ligand and Ti^4+^ ions are formed [7]. Moreover, titanium ions are preferred to form Ti-oxo clusters or infinite Ti-oxo chains/sheets, which will be coordinated with many ligands, further strengthening the stability of Ti-MOFs. However, due to the high reactivity and harsh synthetic conditions of titanium precursors, only a limited number of Ti-MOFs have been reported so far [31,32,33,34,35,36,37,38,39,40,41,42,43,44,45].

**Figure 1:**
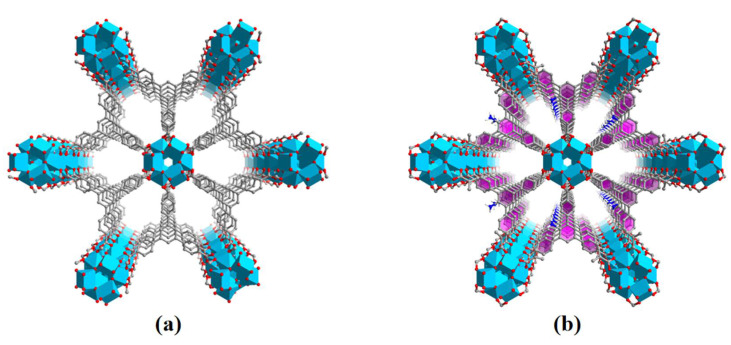
Crystal structures of the ZSTU-2 (**a**) and NH_2_-ZSTU-2 (**b**).

Among the various semiconductors, TiO_2_ is the first example used for photocatalytic hydrogen production due to its light sensitive Ti ions [46]. Superior to TiO_2_, Ti-MOFs not only possess Ti-oxo clusters or Ti-oxo chains/sheets, but also have light harvested ligands, endowing them with promising photocatalytic activity [47]. Especially, the adjustable structures of Ti-MOFs make them efficiently utilize the solar light beyond ultraviolet region (accounts only 4%). Herein, we synthesized an amino functionalized Ti-MOF, named NH_2_-ZSTU-2 (ZSTU stands for Zhejiang Sci-Tech University), for photocatalytic hydrogen production. This MOF is composed of infinite Ti-oxo chains and amino functionalized ternary carboxylic acid ligands, which is isomorphic to ZSTU-2 (Figure 1). Compared with the counterpart ZSTU-2, NH_2_-ZSTU-2 showed a nearly 2.5 times higher photocatalytic hydrogen production activity with a rate of 431.45 μmol·g^−1^·h^−1^.

## 2. Results and Discussions

### 2.1. Structural Characterizations of Photocatalysts

**Figure 2:**
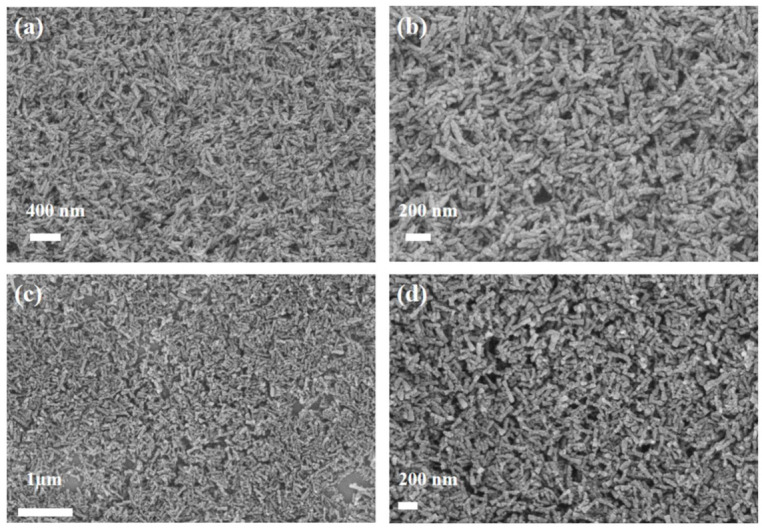
SEM images of ZSTU-2 (**a**,**b**) and NH_2_-ZSTU-2 (**c**,**d**).

**Figure 3:**
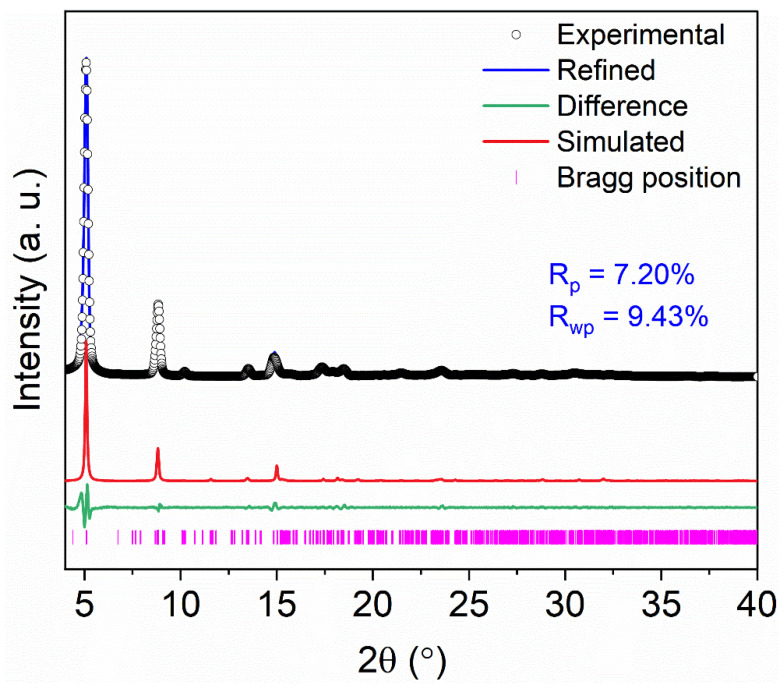
The PXRD analysis of NH_2_-ZSTU-2 displaying the experimental pattern (black circles), refined pattern based on Pawley refinement (blue line), the difference plot (green line), the simulated plot (red line), and Bragg positions (pink).

**Figure 4:**
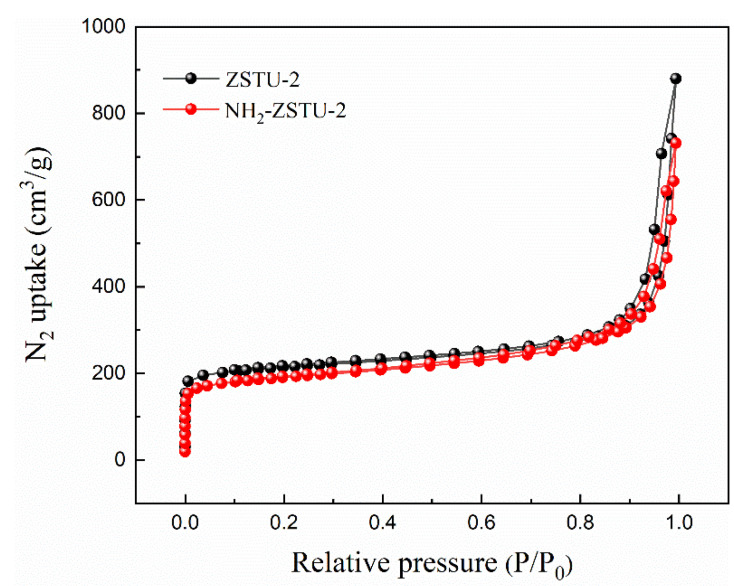
Nitrogen sorption isotherms of ZSTU-2 and NH_2_-ZSTU-2.

The crystallinity of NH_2_-ZSTU-2 was improved by introducing acetic acid as the modulator, which can delay the crystallization speed of MOF, and finally obtain better crystallinity. The regular rod-shaped crystallites with diameter of approximately 50 nm and length of 150 nm were characterized by scanning electron microscope (SEM), which is isomorphic to ZSTU-2 (Figure 2). The size of NH_2_-ZSTU-2 is too small to directly determine the crystal structure using single-crystal diffraction measurements. Therefore, powder X-ray diffraction (PXRD) analysis was used to discovery the MOF structure. The PXRD pattern of NH_2_-ZSTU-2 is quite similar to ZSTU-2 (Figure S1), and we thus modeled the structure of NH_2_-ZSTU-2 using the framework of ZSTU-2 with installed amino group on BTB linkers, followed by structural optimization using material studio. Based on the structure model, Pawley refinement was performed on the PXRD data, and we obtained the unit cell parameters of a = 11. 7987 Å, b = 34.6036 Å, and c = 20.1266 Å, and *α* = *β* = *γ* = 90°, with agreement factors of *R_p_* = 0.0720 and *R_wp_* = 0.0943 for NH_2_-ZSTU-2 (Figure 3), strongly supporting its validity. Detailed lattice parameters and atomic coordinates of NH_2_-ZSTU-2 are provided in Tables S1 and S2. Based on the structure of NH_2_-ZSTU-2 we obtained by Pawley refinement, every six titanium atoms form a secondary unit of Ti_6_(µ_3_-O)_6_(COO)_6_ through a bridge, while such a Ti_6_ cluster is interconnected on the c-axis by adjacent µ_2_-OH to form an infinite one-dimensional [Ti_6_(µ_3_-O)_6_(µ_3_-OH)_6_(COO)_6_]_n_ chain of titanium-oxygen clusters. The 1D Ti-oxo chains were then extended by the triangular NH_2_-BTB linkers to form a 3D porous structure. The high porous structure of NH_2_-ZSTU-2 was further studied by nitrogen sorption isotherms (Figure 4). The calculated BET specific surface area from nitrogen sorption isotherms is about 604 m^2^/g, which is comparable to its counterparts ZSTU-2 (657 m^2^/g). Through the infrared (IR) spectrogram (Figure S2), we can find that the titanium oxide bonds had been formed in both NH_2_-ZSTU-2 and ZSTU-2, with the corresponding vibration band near 773 cm^−1^ [38]. Furthermore, IR vibration band at approximately 1430 cm^−1^, 1604 cm^−1^, 3459 cm^−1^ are associated with the C-O stretching vibration, the benzene ring skeleton vibration, and the stretching vibration of hydroxyl coordination, respectively. Compared with ZSTU-2, an extra vibration band near 3399 cm^−1^ of NH_2_-ZSTU-2 can be attributed to the uncoordinated amino group. In addition, we found that the IR peak at 3399 cm^−1^ was kept but 3459 cm^−1^ was decreased after heating the NH_2_-ZSTU-2 at 200 °C for 2 h under vacuum, which indicated that the absorbed water was vapored and the amino groups were retained after heating. In order to obtain the thermal stability of the MOFs, thermogravimetry analysis (TG) was further studied (Figure S3). Through the TG curves, we can conclude that both NH_2_-ZSTU-2 and ZSTU-2 can maintain their structure at about 400 °C. The weight loss at around 200 °C is mainly attributed to the loss of coordinated solvents in MOFs.

**Figure 5:**
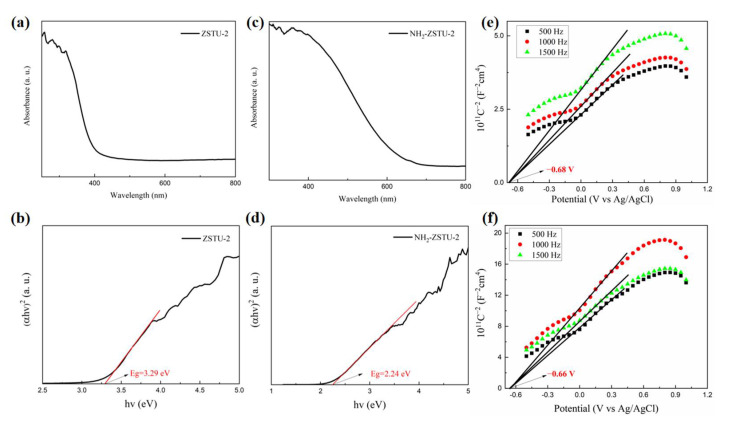
Band structural information of photocatalysts. The solid-state diffuse reflectance absorption spectra of ZSTU-2 (**a**) and NH_2_-ZSTU-2 (**c**); Tauc plots for ZSTU-2 (**b**) and NH_2_-ZSTU-2 (**d**), presenting band gap of MOFs calculated under the hypothesis that absorption follows: (αhν)^2^ = K (hν − E_g_); Mott–Schottky plots of ZSTU-2 (**e**) and NH_2_-ZSTU-2 (**f**).

As we know, the band structures determine thermodynamics of photocatalysts for photocatalytic hydrogen production. The band gaps of ZSTU-2 and NH_2_-ZSTU-2 were first studied by solid-state diffuse reflectance absorption spectra. As shown in Figure 5a, the light harvesting region of ZSTU-2 can only reach 450 nm, and the corresponding band gap calculated from Tauc plot is 3.29 eV (Figure 5b). To extend the absorption range of ZSTU-2 to visible region, amino functionalized NH_2_-BTB linkers were adopted to replace the H_3_BTB linkers during MOF synthesis. The light absorption region of the NH_2_-ZSTU-2 illustrated by solid-state diffuse reflectance absorption spectra can be largely extended to 700 nm (Figure 5c), and the band gap is only 2.24 eV (Figure 5d). For photocatalysts, the larger light-harvesting region and lower band gap mean that they can utilize more sunlight and achieve better photocatalytic hydrogen production performance. The conduction band positions of ZSTU-2 and NH_2_-ZSTU-2 were further determined by Mott–Schottky measurements. Positive slope in both Figure 5e,f indicated that both ZSTU-2 and NH_2_-ZSTU-2 are n-type semiconductors. The conduction band potentials of them were determined to be −0.68 eV and −0.66 eV, respectively. Then the valence band potentials of them were calculated to be 2.61 eV and 1.58 eV, respectively. The energy band diagram of ZSTU-2 and NH_2_-ZSTU-2 are shown in Figure S4. The introduction of amino groups in MOFs mainly shifts the valence band potential to a higher position and shows little impact on the conduction band potential. Based on the band structural information, we can conclude that both ZSTU-2 and NH_2_-ZSTU-2 were favorable for photocatalytic hydrogen production.

### 2.2. Photoelectrochemical Characterizations of Photocatalysts

**Figure 6:**
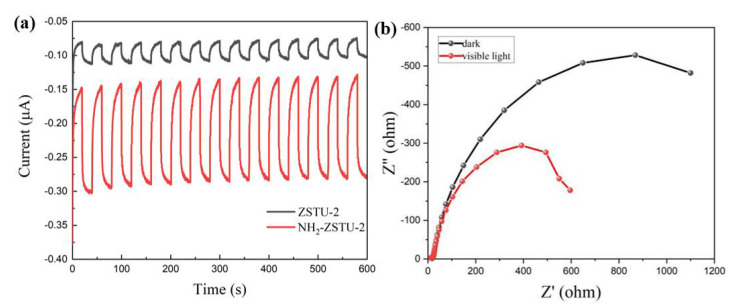
Photoelectrochemical characterizations of photocatalysts. Transient photocurrent plots of NH_2_-ZSTU-2 and ZSTU-2 (**a**); EIS curves of NH_2_-ZSTU-2 under dark and visible light (**b**).

The generation of separated electron-hole pairs was characterized by both transient photocurrent responses and electrochemical impedance spectroscopy (EIS) measurements. As shown in Figure 6a, ZSTU-2 showed low transient photocurrent response under visible light due to the narrow light-harvesting region. As expected, the transient photocurrent responses of NH_2_-ZSTU-2 increased dramatically, which indicated that a better photogenerated charge carries separation efficiency. The EIS of NH_2_-ZSTU-2 was further studied both with and without visible light irradiation. As shown in Figure 6b, compared with the dark state, dramatically decreased radius of the EIS curve under visible light irradiation indicated that a large number of separated electron-hole pairs were photogenerated in NH_2_-ZSTU-2 with visible light shining on.

### 2.3. Photocatalytic Hydrogen Production of Photocatalysts

**Figure 7:**
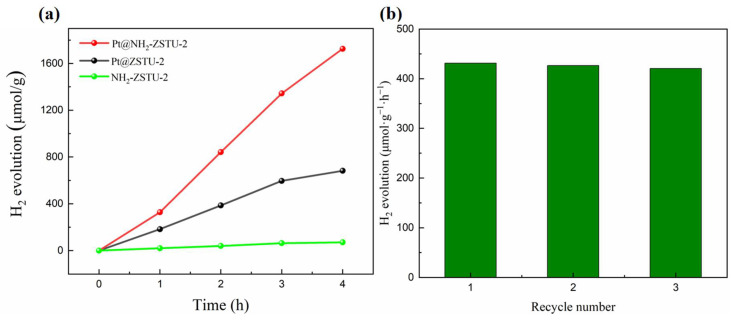
Photocatalytic performance. (**a**) Time-dependent photocatalytic hydrogen production of Pt@ZSTU-2, Pt@NH_2_-ZSTU-2, and NH_2_-ZSTU-2 in triethanolamine/acetonitrile/water system under visible light irradiation; (**b**) recycle performance of Pt@NH_2_-ZSTU-2 under same condition.

Before photocatalytic hydrogen production, both MOFs were loaded with Pt using a photo deposition method [48]. The Pt nanoparticles were successfully deposited in MOFs and characterized by TEM (Figure S5). The photocatalytic hydrogen production was then performed in TEOA/CH_3_CN/H_2_O mixed solvents under 300 W Xe lamp irradiation with a L42 light filter and triethanolamine (TEOA) as a sacrificial agent, and Pt as cocatalyst [48]. Before the photocatalytic reaction, the solution was degassed for 20 min to remove the dissolved O_2_ in solvent. The production of hydrogen was detected by an on-line GC with a TCD detector. As shown in Figure 7a, both Pt@ZSTU-2 and Pt@NH_2_-ZSTU-2 showed steady hydrogen production rate under visible light irradiation. The average photocatalytic H_2_ yields of Pt@ZSTU-2 and Pt@NH_2_-ZSTU-2 were 170.45 μmol·g^−1^·h^−1^ and 431.45 μmol·g^−1^·h^−1^, respectively. The almost 2.5 times enhanced photocatalytic hydrogen production rate of Pt@NH_2_-ZSTU-2 is mainly attributed to the enlarged light-harvested region. It should be noted that the cocatalyst Pt plays important role on photocatalytic hydrogen production. The hydrogen production rate of Pt@NH_2_-ZSTU-2 is also comparable to the state-of-the-art Ti-MOFs, such as PCN-416 (484 μmol·g^−1^·h^−1^), MIL-100(Ti) (42 μmol·g^−1^·h^−1^), MUV-10(Mn) (271 μmol·g^−1^·h^−1^), NH_2_-MIL-125 (367 μmol·g^−1^·h^−1^) [28,49,50,51]. The stability of Pt@NH_2_-ZSTU-2 during photocatalysis was studied by the recycle experiments, which indicated that Pt@NH_2_-ZSTU-2 is stable at least three cycles under visible light irradiation. The hydrogen evolution rates of the first, second and third cycles were 431.45 μmol·g^−1^·h^−1^, 421.50 μmol·g^−1^·h^−1^ and 420.71 μmol·g^−1^·h^−1^, respectively (Figure 7b). The retained PXRD patterns of the recycled Pt@NH_2_-ZSTU-2 also indicated that Pt@NH_2_-ZSTU-2 is stable (Figure S3).

**Figure 8:**
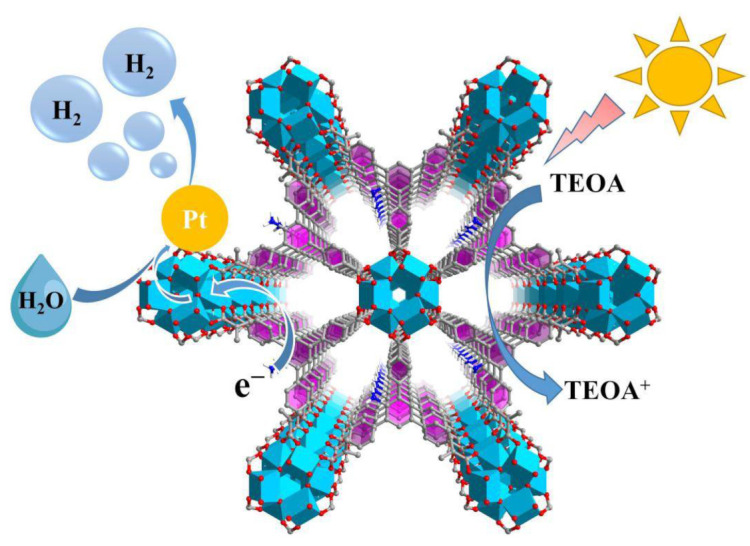
Proposed photocatalytic hydrogen production mechanism over NH_2_-ZSTU-2 under visible light irradiation.

A proposed, photocatalytic hydrogen evolution mechanism of Pt@NH_2_-ZSTU-2 is shown in Figure 8. Under visible light irradiation, NH_2_-BTB linkers absorb light and the generated photogenerated electrons then transfer to infinite Ti-oxo chains through LMCT mechanism, thus reducing Ti^4+^ to Ti^3+^, and the photogenerated electrons in NH_2_-ZSTU-2 conduction band transfer to Pt cocatalyst for reduction of water to produce H_2_ [16,26,35]. The holes in the valence band oxidize the sacrificial agent TEOA to TEOA^+^, constituting a complete REDOX reaction.

## 3. Experimental

### 3.1. Synthesis of NH_2_-ZSTU-2

2,4,6-tris(4-carboxyphenyl)-aniline (NH_2_-BTB) (100 mg, 0.220 mmol) and ultra-dry DMF (5 mL) were first added into a 25 mL Teflon-lined stainless-steel autoclave, and then 100 μL glacial acetic acid was added dropwise. After sonication for 10 min, NH_2_-BTB was fully dissolved to obtain a yellow transparent solution, and then titanium tetraisopropanolate (Ti(i-Pro)_4_) (0.04 mL, 0.128 mmol) was added dropwise, and sonication was performed for 20 min to form a yellow slurry. The autoclave was then heated in an oven at 190 °C for 22 h. After cooling down, the yellow powder NH_2_-ZSTU-2 was obtained by centrifuging and washing with DMF and methanol for several times. At last, NH_2_-ZSTU-2 was dried in a vacuum oven at 60 °C for 12 h to remove the residual methanol. CHN element analysis data of NH_2_-ZSTU-2 had also been done with average weight ratio of 43.915:2.612:2.18. The chemical formula of NH_2_-ZSTU-2 was determined to be Ti_6_(μ_3_-O)_6_(μ_2_-OH)_6_(NH_2_-BTB)_2_ (DMF)_0.3_ based on element analysis and its structural information obtained from Pawley refinement of PXRD data.

### 3.2. Synthesis of Pt@NH_2_-ZSTU-2

Pt NPs were deposited in the NH_2_-ZSTU-2 using a photo deposition method [52]. First, NH_2_-ZSTU-2 (50 mg) was dispersed in a mixture of H_2_O (8 mL) and MeOH (13 mL) in a reaction vessel. After NH_2_-ZSTU-2 was fully dispersed in the mixture, 1 mL chloroplatinic acid hexahydrate aqueous solution (1.33 mg·mL^−^^1^) was then added and the system was vacuumed for 20 min to remove the air. The mixture was then irradiated with a 300 W Xe lamp without light filter for 4 h. The sample was then centrifuged and dried overnight in an oven at 100 °C, and resulted sample was labeled as Pt@NH_2_-ZSTU-2. The chlorine and Pt content in NH_2_-ZSTU-2 had been determined to be 1.25 wt% and 9.33 wt% by energy dispersive spectrometer (FESEM, JEOL, Japan).

### 3.3. Photoelectrochemical Measurementsz

Electrode Preparation: About 10 mg of photocatalyst was dispersed in 1 mL of isopropanol, and then 30 μL of naphthol solution (5% *w*/*w* in water) was added, and the mixture was sonicated for 2 h afterwards. The obtained dispersion was then dropped onto one side of a FTO glass with an area of 1 × 1 cm^2^ (total area of 1 × 3 cm^2^), and dried in air at 60 °C on a hotplate.

Photocurrent measurements were carried out on an electrochemical workstation (Shanghai Chenhua Instrument Co. Ltd., Shanghai, China) in a standard three-electrode system with photocatalysts-coated FTO as the working electrode, Pt net as the counter electrode, Ag/AgCl as the reference electrode, and 0.5 M Na_2_SO_4_ solution (pH ≈ 7.0) as the electrolyte. A 300 W Xe lamp with a L42 light filter was used as visible light source, and the photo-responsive signals of photocatalysts was then recorded with alternating 20 s light on/off. Mott–Schottky plots of those photocatalysts were also performed on the same workstation in a standard three-electrode system at frequencies of 500, 1000, 1500 HZ. EIS curves were obtained using the same workstation and photocatalysts-coated FTO was used as the working electrode.

### 3.4. Photocatalytic Hydrogen Production Experiments

The photocatalytic hydrogen production experiments were evaluated using a batch-type reaction system (Beijing Perfectlight Technology, Beijing, China) at ambient temperature irradiated by a 300 W Xe lamp equipped with a UV cut-off filter (>420 nm). The temperature of condensed circulating water for cooling down the solvent vapor was set to 1 °C. In a typical procedure, 50 mg sample was dispersed into 102 mL mixed solution of acetonitrile, triethanolamine (TEOA), and de-ionized water with volume ratio of 9:1:0.2, and then the suspension was vacuumed for 10 min to remove air. Hydrogen gas was measured by an on-line gas chromatography (GC) (Techcomp-GC7900, argon as a carrier gas) using a thermal conductivity detector (TCD). The production of hydrogen was quantified by a calibration plot to the internal hydrogen standard. For the recycle experiment, the procedure is as follows: after the first experiment test, the system was vacuumed to remove the produced hydrogen and then the second run was restarted the next day. Same procedure was carried out for the third run. In this way, we can avoid the loss of photocatalyst during recovery.

## 4. Conclusions

In this work, we had synthesized an amino-functionalized Ti-MOF, named NH_2_-ZSTU-2, for photocatalytic hydrogen production. The NH_2_-ZSTU-2 was synthesized by a facile solvothermal method, composed of 2,4,6-tri(4-carboxyphenylphenyl)-aniline (NH_2_-BTB) triangular linker and infinite Ti-oxo chains. The structure of NH_2_-ZSTU-2 was fully studied by PXRD, SEM, nitrogen sorption isotherms, etc. The band structural information was also obtained by using solid-state diffuse reflectance absorption spectra and Mott-Schottky measurements, which conclude that NH_2_-ZSTU-2 was favorable for photocatalytic hydrogen production. The generation of separated electron-hole pairs was also characterized by both transient photocurrent responses and electrochemical impedance spectroscopy (EIS) measurements, further showing the potential photocatalytic hydrogen production ability of NH_2_-ZSTU-2. Benefitting from those structural features, NH_2_-ZSTU-2 showed steady hydrogen production rate under visible light irradiation with average photocatalytic H_2_ yields of 431.45 μmol·g^−1^·h^−1^ with triethanolamine and Pt as sacrificial agent and cocatalyst, respectively, which is almost 2.5 times higher than that of its counterpart ZSTU-2. The stability and proposed photocatalysis mechanism were also discussed. This work paves the way to design Ti-MOFs for photocatalysis.

## Data Availability

Not applicable.

## Supplementary Materials

The following supporting information can be downloaded at: https://www.mdpi.com/article/10.3390/molecules27134241/s1, Figure S1: The PXRD patterns of the simulated ZSTU-2, as synthesized ZSTU-2, and NH2-ZSTU-2; Figure S2: Infrared spectra of the ZSTU-2, NH2-ZSTU-2, and NH2-ZSTU-2 after heating at 200 ℃ under vacuum; Figure S3: Thermogravimetry analysis of ZSTU-2 and NH_2_-ZSTU-2 under nitrogen atmosphere; Figure S4: The energy band diagram of the ZSTU-2 and NH_2_-ZSTU-2; Figure S5: TEM image of Pt@NH2-ZSTU-2; Figure S6: Powder XRD patterns of NH_2_-ZSTU-2 before and after three cycles of photocatalytic hydrogen production; Table S1: Crystal data and refinement details; Table S2: Fractional atomic coordinates.
