# Supplementary material for: Amino-Functionalized Titanium Based Metal-Organic Framework for Photocatalytic Hydrogen Production
Source: Molecules. 2022 Jun 30;27(13):4241. doi: 10.3390/molecules27134241 (PMC9268624; doi:10.3390/molecules27134241)
Supplement: Supplementary file 1 [file molecules-27-04241-s001.zip › molecules-1787526-supplementary.pdf]

Amino-Functionalized Titanium based Metal-Organic Framework for Photocatalytic Hydrogen Production

Niannian Hu[1], Youlie Cai[1], Lan Li[3], Xu-Sheng Wang[1,2,4]* and Junkuo Gao[1,2]*

[1]Institute of Functional Porous Materials, School of Materials Science and Engineering, Zhejiang Sci-Tech University, Hangzhou 310018, China

[2]Zhejiang Provincial Innovation Center of Advanced Textile Technology, Shaoxing, 312000, China

[3] College of Materials Science and Engineering, China Jiliang University, Hangzhou 310018, China

[4] College of Chemistry and Materials Science, Jinan University, Guangzhou 510632, China

*Correspondence: xswang@zstu.edu.cn (X. S. W.); jkgao@zstu.edu.cn (J. G.)

1. Chemicals.

4,4',4''-benzene-1,3,5-tribenzoic acid (BTB) (Purity 98%, Shanghai Kaishu Chemical Technology), 2,4,6-tri(4-carboxyphenylphenyl)aniline (NH_2-BTB) (Purity 98% Nanchang Chouhe Pharmaceutical Technology), DMF (Purity 99.8%, Annegie Chemical Reagents), Methanol (Analysis Pure, Aladdin Chemical Reagents), Isopropyl titanate (Ti analyzes pure, TCI chemical), Chloroplatinic acid (H_2PtCl_6) (assay pure, Aladdin Chemical Reagents), Ethanol (analysis pure, Hangzhou Gaojing Fine Chemical), Isopropanol (analysis pure, Hangzhou Gaojing Fine Chemical), Glacial Acetic Acid (Analytical Pure, Aladdin Chemical Reagents), Acetonitrile (Analytical Pure, Aladdin Chemical Reagents), Triethanolamine (Analytical Pure, Aladdin Chemical Reagents).

2. Material characterization

Field emission scanning electron microscopy (SEM) (JSM-6700F, FESEM, JEOL, Japan) was used to observe the microscopic morphology of MOFs with an acceleration voltage of 3 kV.Transmission electron microscopy (TEM) image were obtained by JEM-2100. The crystal structure of MOFs was tested using a powder X-ray diffractometer (PXRD, Bruker AXS D8 Advance) with Cu target Ka radiation at a scanning range of 3° to 40° and a scanning rate of 3° min^{-1}. The surface area and porosity of MOFs were were characterized using the Specific Surface Area Analyzer (3H-2000PS). Fourier transform infrared spectra were recorded from blank KBr pellets using the Perkin Elmer FTIR SpectrumGX spectrometer. Thermogravimetric analysis (TGA) data were collected from TGA/DSC 2 thermogravimetric analyzer (Mettler-Toledo Corp. Switzerland) in N_2 atmosphere at a heating rate of 10 ℃/min up to 800 ℃. The UV-VIS absorption spectra of ZSTU-2 and NH_2-ZSTU-2 were measured by Shimadzu UV-2450 spectrophotometer. Barium sulfate was used as a reference material. Using the Tauc plot method:

$$(\alpha h\upsilon)^2=K(h\upsilon-Eg) \quad (1)$$

Here "α" is the absorption coefficient, "hυ" is the incident photon energy, "K" is an energy independent constant, "Eg" is the bandgap energy. Let's start from the Absorbance data and apply Tau's equation (1) to check its bandgap energy.

Using equation (1), we will plot:

hυ=1240/wavelength On x-axis and $(\alpha h\upsilon)^2$ On Y-axis.

3. Synthesis of ZSTU-2

4,4',4"-benzene-1,3,5-tribenzoic acid (H_3BTB) (90 mg, 0.205 mmol) and DMF (5 mL) was firstly added into a 25 mL Teflon-lined stainless-steel autoclave, and then 100 μL glacial acetic acid was added dropwise. After sonication for 10 min, H_3BTB was fully dissolved to obtain a transparent solution, and then $Ti(i\text{-}OPr)_4$ (0.04 mL, 0.128 mmol) was added dropwise, and sonication was performed for 20 min to form a white slurry. The autoclave was then heated in an oven at 190 °C for 22 h. After cooling down, the yellow powder ZSTU-2 was obtained by centrifuging and washing with DMF and methanol for several times. At last, ZSTU-2 was dried in a vacuum oven at 60 °C for 12 h to remove the residual methanol.

4. Refinement of NH_2-ZSTU-2

Since the PXRD pattern of NH_2-ZSTU-2 are highly similar to that of ZSTU-2, the primary structure of NH_2-ZSTU-2 was obtained through installing amino group onto ZSTU-2 structure and optimized in Material Studio 8.0. Then, the Pawley refinement of the experimental PXRD was conducted by the Reflux module in the Material Studio 8.0. Crystal data and refinement details were list in the Table S1-2.

5. Additional figures and tables

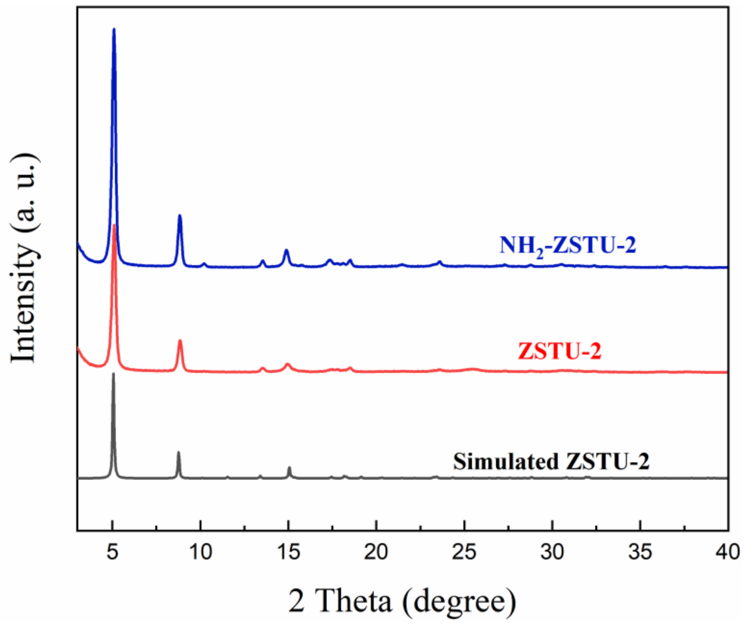

Figure S1. The PXRD patterns of the simulated ZSTU-2, as synthesized ZSTU-2, and NH_2-ZSTU-2.

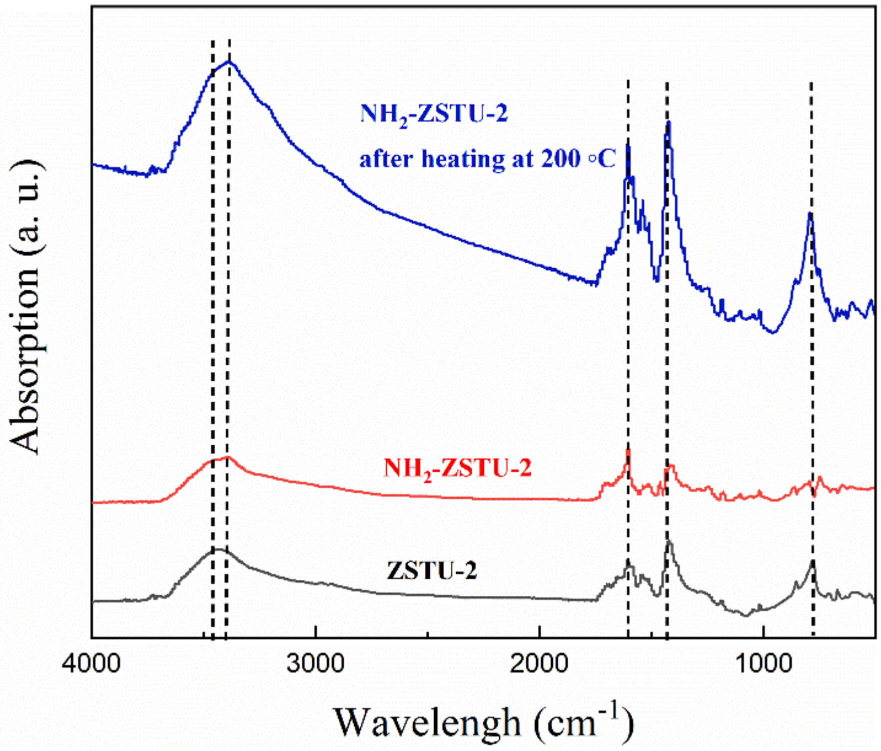

Figure S2. Infrared spectra of the ZSTU-2, NH_2-ZSTU-2, and NH_2-ZSTU-2 after heating at 200 °C under vacuum.

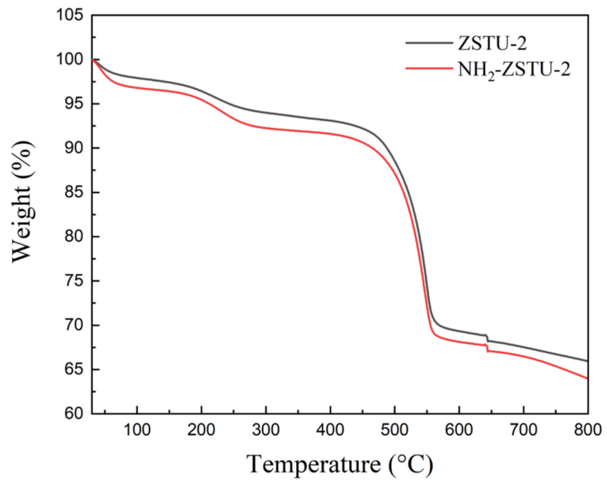

Figure S3. Thermogravimetry analysis of ZSTU-2 and NH_2-ZSTU-2 under nitrogen atmosphere.

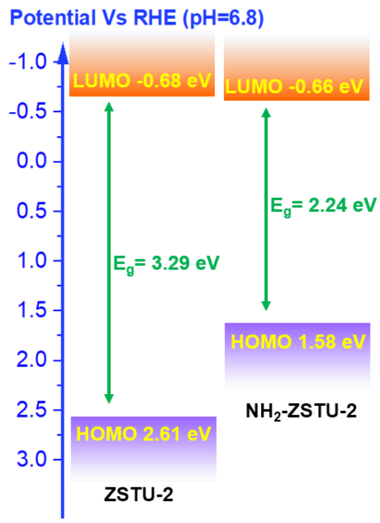

Figure S4. The energy band diagram of the ZSTU-2 and NH_2-ZSTU-2.

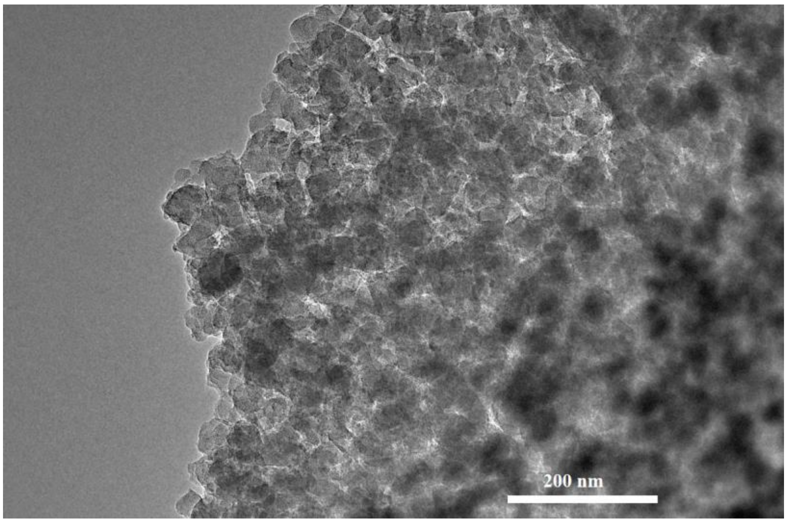

Figure S5. TEM image of Pt@NH_2-ZSTU-2.

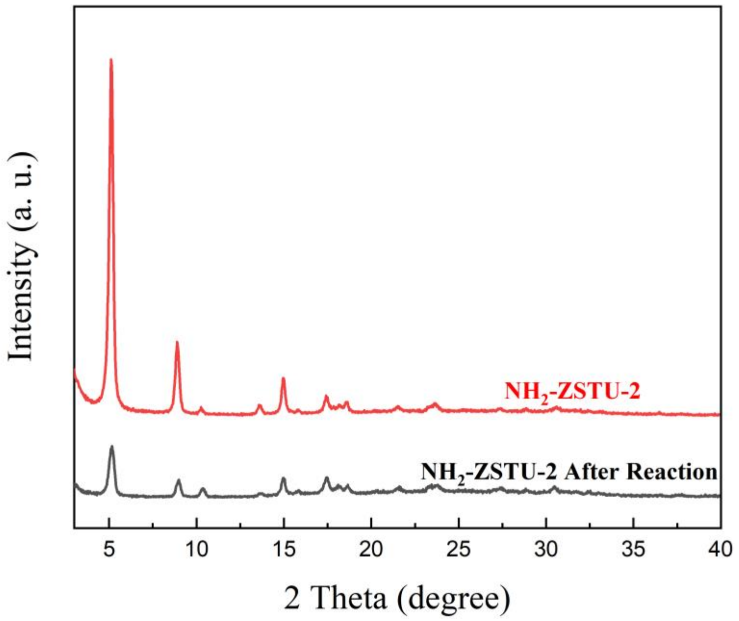

Figure S6. Powder XRD patterns of NH_2-ZSTU-2 before and after three cycles of photocatalytic hydrogen production.

Table S1. Crystal data and refinement details.

Structure name	NH_2-ZSTU-2
CCDC number	2182550
Crystal system	orthorhombic
Space group	Amm2
a/Å	11.7987
b/Å	34.6036
c/	20.1266
α (°)	90.000
β (°)	90.000
γ (°)	90.000
Unit cell volume/$Å^3$	8217.24
Z	4
Temperature/K	293
Wavelength/Å	1.54051
R_p	0.0720
R_{wp}	0.0943

Table S2. Fractional atomic coordinates.

Atom name	*x*	*y*	*z*
O1	0.68000	0.96640	0.81748
C6	0.68440	0.90991	0.63282

C7	0.68351	0.92987	0.69286
H6	0.63233	0.92000	0.59143
H7A	0.63187	0.95577	0.69937
O10	0.68000	0.60823	0.53888
C26	0.68440	0.72950	0.54710
C25	0.68351	0.68926	0.54681
H26	0.63233	0.74530	0.58283
H25	0.63187	0.67304	0.58212
O5	0.68000	0.92765	0.14573
C15	0.68440	0.86288	0.32216
C16	0.68351	0.88314	0.26241
H15A	0.63233	0.83697	0.32782
H16	0.63187	0.87346	0.22059
O6	0.81828	0.96639	0.18419
C19	0.81387	0.90991	0.36885
C18	0.81476	0.92987	0.30881
H19	0.86594	0.92000	0.41024
H18	0.86640	0.95577	0.30228
O9	0.81828	0.60823	0.46278
C22	0.81387	0.72950	0.45456
C23	0.81476	0.68926	0.45485
H22	0.86594	0.74530	0.41883
H23	0.86640	0.67304	0.41955
O2	0.81828	0.92764	0.85594
C4	0.81387	0.86288	0.67950
C3	0.81476	0.88314	0.73925
H4	0.86594	0.83697	0.67384
H3A	0.86640	0.87346	0.78107
O31	0.82186	0.54041	0.54279
Ti3	0.64463	0.54976	0.55206
C5	0.74914	0.87595	0.62509
C2	0.74914	0.91677	0.74662
C1	0.74914	0.93839	0.81100
C8	0.74914	0.85455	0.56134
C21	0.74914	0.75036	0.50041
C24	0.74914	0.66874	0.50041
C27	0.74914	0.62550	0.50041
C10	0.74914	0.79318	0.50041
C14	0.74914	0.87595	0.37657

C17	0.74914	0.91677	0.25505
C20	0.74914	0.93839	0.19066
C12	0.74914	0.85455	0.44032
C13	0.74914	0.87445	0.50041
H13	0.74914	0.90602	0.50041
C9	0.74914	0.81392	0.56051
H9	0.74914	0.79813	0.60751
C11	0.74914	0.81392	0.44115
N1	0.75202	0.79185	0.37637
H1B	0.71311	0.76620	0.37026
H1A	0.80171	0.80396	0.34222
O14	0.50000	0.57025	0.51064
H14	0.50000	0.58553	0.47108
O7	1.00000	0.97116	0.11350
H7	1.00000	0.94318	0.11606
O8	0.67642	1.00000	0.08516
Ti2	0.85365	1.00000	0.10371
O13	0.67642	0.54041	0.45887
Ti4	0.85365	0.54976	0.44960
O15	0.50000	0.53914	0.60329
H15	0.50000	0.52643	0.64623
O4	0.82186	1.00000	0.91650
Ti1	0.64463	1.00000	0.89795
O11	1.00000	0.57025	0.49102
H11	1.00000	0.58553	0.53058
O12	1.00000	0.53914	0.39837
H12	1.00000	0.52643	0.35543
O3	0.50000	0.97116	0.88816
H3	0.50000	0.94318	0.88560
